# Sericin-Enriched Rabbit Semen Preservation: Implications for Short-Term Storage Quality and Fertility at 4 or 15 °C

**DOI:** 10.3390/ani14233429

**Published:** 2024-11-27

**Authors:** Sanan Raza, Uğur Uçan, Melih Aksoy, Güneş Erdoğan, Zahid Naseer, Komal Khan

**Affiliations:** 1 Department of Clinical Sciences, Theriogenology Section, University of Veterinary and Animal Sciences, Sub-Campus, Jhang 35200, Pakistan; 2 Department of Reproduction and Artificial Insemination, Faculty of Veterinary Medicine, Aydin Adnan Menderes University, Aydın 09016, Türkiye; ugur.ucan@adu.edu.tr (U.U.); melih.aksoy@adu.edu.tr (M.A.); 3 Department of Veterinary Obstetrics and Gynecology, Faculty of Veterinary Medicine, Aydin Adnan Menderes University, Aydın 09016, Türkiye; gerdogan@adu.edu.tr; 4 Department of Clinical Studies, Faculty of Veterinary and Animal Sciences, Pir Mehr Ali Shah Arid Agriculture University, Rawalpindi 46000, Pakistan; 5 Department of Basic Sciences, Anatomy Section, University of Veterinary and Animal Sciences, Sub-Campus, Jhang 35200, Pakistan; komal.khan@uvas.edu.pk

**Keywords:** acrosome, chilled storage, fertility, motility, rabbit sperm, sericin

## Abstract

The influence of sericin supplementation and chilling temperatures (4 and 15 °C) on the short-term storage quality and fertility of rabbit semen was assessed over 72 h of storage. In experiment 1, pooled semen (five replications) was diluted to a concentration of 50 M mL^−1^, and assigned to control, 0.1%, and 0.5% sericin groups, stored at 4 or 15 °C. Sperm motility, sperm kinematics, viability, and membrane and acrosome integrity were assessed at 0, 24, 48, and 72 h of storage. Experiments 2 and 3 were conducted to observe the effect of sericin on bacterial growth and in vivo fertility. The results of experiment 1 showed that sericin treatment, storage temperature, and storage duration influenced progressive and total sperm motility. The storage duration affected all sperm kinematics variables, whereas VCL, VSL, VAP, and BCF sperm kinematics altered in response to sericin treatment and storage temperature. Similarly, significant effects of sericin treatment, storage temperature, and storage duration were observed for acrosome integrity, sperm membrane integrity, and sperm viability. The results of experiment 2 indicated the antimicrobial effects of sericin when sperm were stored at 15 °C for 72 h. While promising pregnancy outcomes were observed with sericin-treated sperm for 72 h, these results were not significantly different. This study conclusively demonstrates that sericin treatment enhances the quality of rabbit semen when stored at lower temperatures for longer durations. The antimicrobial effects of sericin could be a contributing factor to the improved in vitro and in vivo fertility of rabbit sperm.

## Simple Summary

Sericin, a protein derived from silk, is used for different biological functions such as oxidation resistance and antimicrobial activity. It has promising potential in preserving sperm quality when used as a supplement during semen refrigeration or cryopreservation. Due to its antioxidant and antimicrobial properties, sericin helps protect sperm cells from oxidative stress and bacterial contamination, which can otherwise impair sperm quality. When added to stored semen, sericin has been found to improve key sperm characteristics such as motility, viability, and membrane integrity, all of which are crucial for successful fertilization. Sericin’s preservation of sperm quality highlights its potential as a valuable tool in reproductive biotechnology for extending the viability and function of stored semen.

## 1. Introduction

Cryopreservation and chilling are the predominant strategies for sperm storage in various species, each with inherent advantages and limitations. Although cryopreservation offers extended storage durations, it severely affects sperm motility, viability, acrosomal integrity, and fertilizing capacity [1]. Accordingly, the success rates of rabbit sperm cryopreservation vary with the use of different proposed extenders, e.g., DMSO [1], dextran [2], luteolin [3], and curcumin [4].

Lower conception rates of frozen semen compared to fresh semen present a significant challenge in achieving optimal pregnancy rates in rabbits, necessitating strategies to protect sperm against cryo-injuries [5]. Daniel and Renard [6] also advocated for reliance on fresh semen AI in rabbits, as suboptimal fertility rates with frozen semen present a significant bottleneck at an industrial level. Additionally, Di Iorio et al. [7] have reported promising fertility rates of frozen rabbit semen, although rabbit semen freezing is a costly technique Di Iorio et al. [8]. Conversely, chilling semen at 4 or 15 °C offers a simpler and more cost-effective option for AI in the rabbit industry, though it is not without challenges. Storing semen at higher temperatures (5 to 25 °C) has its potential benefits, including ease of dilution, storage, transport, and efficient revival of sperm function in field conditions. Mocé et al. [9] documented higher pregnancy rates with rabbit semen chilled from 4 to 22 °C, which is more practical in field conditions. The primary concern in chilling rabbit semen is the production of reactive oxygen species (ROS) and bacterial proliferation [2]. The use of antibiotics in chilled semen also raises concerns over antibiotic resistance, which urges the exploration of alternative extenders for sperm storage [2,10]. Previous studies have investigated the use of l-carnitine [11], honey [12], and melatonin [13] to protect rabbit sperm in different methods of freezing. During the chilling of rabbit sperm, various alternative agents have been included as an extender to protect sperm under storage conditions by supplying nutrients for energy, pH modulation, cold-shock resistance, antioxidants, and bacterial growth inhibitors [14]. In addition to these, chilling temperatures also affect bacterial growth and sperm metabolism [15], which in turn leads to oxidative stress and the premature depletion of energy resources in the semen. To mitigate the adverse effects on sperm during low-temperature storage, sericin, a silk-derived protein, has been utilized as a cryoprotective, antioxidant, and antimicrobial agent [16]. Sericin possesses adhesive properties, is rich in amino acids, and has water solubility along with potential cryoprotective properties for sperm [17] and embryos in rabbits [18], bucks [19], humans [20], boars [21], and roosters [22]. This study aims to investigate the effects of sericin and different storage temperatures on sperm through in vivo and in vitro experiments, offering new insights into alternative preservation strategies for rabbit sperm storage.

## 2. Materials and Methods

### 2.1. Extender Preparation

A basic tris−citric acid−glucose (TCG) extender was prepared for semen dilution, comprising tris (250.04 mM), citric acid (79.76 mM), glucose (69.38 mM), streptomycin (75.00 IU), and penicillin-G (166.20 IU). The pH and osmolarity of the extender were meticulously adjusted to 7.14 and 299 mOsm/kg, respectively. The extenders were immediately stored at −20 °C until use. All the chemicals were procured from Sigma-Aldrich, St. Louis, USA.

### 2.2. Animal Selection and Husbandry Practices

The present study was sanctioned by the Aydin Adnan Menderes University Animal Ethics Committee for the welfare and use of experimental animals (ADÜ-HADYEK No. 64583101/2020/007). For this purpose, 12 (*n* = 12) male New Zealand white rabbits with an average body weight of 2.9 ± 0.1 kg, and aged 10–12 months were selected. The bucks were separately confined in galvanized wire cages (70 × 50 × 35 cm) in a naturally ventilated environment with standard daylight (12–14 h) at a room temperature of 16–28 °C. They were offered commercially prepared feed with free access to clean fresh water. The rabbit bucks underwent proper training for semen collection using teaser does, and only high-quality semen donor bucks were selected for further experiments.

### 2.3. Semen Collection and Evaluation

The semen was collected from rabbit bucks by a single operator using an artificial vagina mounted over a teaser doe. Following collection, the gel plug was immediately discarded, and the semen was placed in a water bath at 37 °C for further evaluation. All individual ejaculates were examined for motility, concentration, and morphology through standard procedures [23]. Ejaculates with ≥70% motile, 200 × 10^6^ mL^−1^, and 80% morphologically normal sperm were selected for further experimentation.

### 2.4. Sperm Treatment and Processing

The best qualifying ejaculates (at least five ejaculates from experimental bucks) after the initial evaluation were pooled (one ejaculate/male; five ejaculates as a pool) and diluted with the TCG extender to achieve a final sperm concentration of 50 × 10^6^ mL^−1^. Afterwards, the diluted semen was split into three aliquots and categorized into control, 0.1, and 0.5% sericin groups (Sericin Bombyx mori-silkworm, S5201, Sigma-Aldrich, St. Louis, MO, USA). Each treatment group was stored at either 4 °C or 15 °C for a period of 72 h. All the samples were observed for sperm motility, sperm kinematics, acrosome integrity, sperm viability, and plasma membrane integrity at 0, 24, 48, and 72 h of the storage period at 4 or 15 °C. The experiment was replicated at least five times for observation recording.

### 2.5. Sperm Motility and Kinematics

The motility assessment of each sample was performed using the CASA system (SCA^®^-Sperm Class Analyzer, Microptic S.L. Viladomat, Barcelona, Spain) connected to a phase-contrast microscope (Olympus, CX41, Tokyo, Japan) with an adjustable heated stage and camera. The semen samples were subjected to a motility assessment using a 3 µL drop placed over a prewarmed glass slide covered with a coverslip (18 × 18 mm^2^). The CASA system specifications were adjusted for rabbit sperm motility properties and standardized accordingly. To detect only the sperm head and exclude other particles or gel droplets, a particle size >10 µm was used. During the analysis, at least five fields were randomly selected on the slide (100 sperms/field at ×10).

### 2.6. Evaluation of Sperm Viability

A small drop of semen and 1% nigrosin–eosin stain (Sigma-Aldrich, St. Louis, MO, USA) were combined over a prewarmed glass slide to prepare thin and uniform smears. The air-dried smears were then observed using phase-contrast microscopy at ×1000 (oil emersion lens) for unstained heads (live sperm) and stained or partially stained heads (dead sperm).

### 2.7. Assessment of Sperm Membrane Integrity

A small volume of semen sample (25 µL) was exposed to a hypo-osmotic solution (475 µL, 100 mOsm/kg) and incubated for 15 min at 35 °C. After the osmotic challenge, the percentage of membrane-intact sperm was calculated by preparing a wet mount. Sperm sub-populations were counted based on the curliness of the sperm tail portion to assess the integrity or damage to the plasma membrane. Approximately 200 sperm populations were counted using a bright-field microscope (40×) (Olympus Corporation, Hachioji, Tokyo, Japan).

### 2.8. Evaluation of Acrosome Integrity

A Coomassie Blue G-250 (Sigma-Aldrich, St. Louis, MO, USA) staining method was adopted from a previous study to assess the percentage of acrosome-intact sperm [24]. Firstly, 50 µL of sperm was fixed for 10 min in a 4% paraformaldehyde solution (110 mM Na_2_HPO_4_, 2.5 mM NaH_2_PO_4_, 4% paraformaldehyde, pH 7.4) at room temperature. The samples were then centrifuged, and the pellet was washed twice using ammonium acetate (100 mM; pH 9.0). Subsequently, a 25–50 µL sperm suspension was transferred onto a glass slide, smeared, and air-dried. Coomassie stain (0.22% Coomassie Blue G-250, 50% methanol, 10% glacial acetic acid, 40% water) was poured over the smeared area on the slide for 2 min. The excess stain was washed off with distilled water, and the cells were counted by using a bright-field microscope at 100× magnification. A total of 200 sperm were counted for each group per replicate.

### 2.9. Assessment of Bacterial Growth

The semen samples stored at 15 °C for 72 h were processed to assess the bacterial load. The samples were homogenized with physiological saline at a 1:9 (*v*/*v*) ratio, and 100 µL of appropriate dilutions was seeded in parallel using plate count agar (Oxoid CM 325, Thermo Scientific™, Waltham, MA, USA) medium by streaking in duplicates. Petri dishes were incubated for 48 h at 36 °C, and the developing colonies were evaluated according to the OIE (1998) protocol [25]. Plates containing between 15 and 300 colony-forming units (CFUs) were counted, while those with >300 CFUs were graded as “+300 UFC”. The CFU mL^−1^ for each sample was calculated by multiplying the average number of colonies counted in duplicates by the inverse of the highest dilution, considering a 100 µL inoculum per plate. All colonies grown on Petri dishes containing plate count agar medium were counted following the procedure described by Gączarzewicz et al. [26], and typical total coliform colonies were considered for culture using violet red bile agar (1–2 mm in diameter, red with a pinkish precipitation halo).

### 2.10. Evaluation of In Vivo Fertility

To observe the effect of sericin-treated sperm stored for 72 h at 4 or 15 °C on fertility, ninety-six New Zealand white rabbit does were inseminated. Receptive multiparous does (3 to 4.5 kg) from the experimental group (32 does per group: control, 0.1%, and 0.5% sericin) with reddened to purple vaginal mucosa were selected and inseminated transvaginally using 12.5 × 10^6^ sperm. The does were held in either dorsal or ventral recumbency, and the semen was transferred to the uterine bifurcation. To induce ovulation, the does were injected with GnRH (Buserin, 0.2 mL = 0.004 mg/mL, i.m, Kayseri, Turkey) at the time of insemination. The pregnancy status of the does was assessed using transabdominal ultrasonography with a micro-convex probe (Mylab 30-Esaote^®^, Genova, Italy) on day 9 post-insemination. During the ultrasound scan, the presence of a fetal heartbeat and gestational sacs were considered positive signs of pregnancy.

Pregnancy rate was calculated using the following formula:

$$Pregnancy\;rate\;(\%)=\frac{Number\;of\;pregnant\;does}{Total\;number\;of\;inseminated\;does}\times 100$$

### 2.11. Statistical Analyses

All the data in this study were analyzed using R-Studio (Boston, MA, USA). In experiment 1, the variables, extender type (control, 0.1, or 0.5% sericin), storage temperature (4 or 15 °C), and storage time (0, 24, 48, or 72 h) were incorporated and subjected to the Generalized Linear Mixed Model (GLMM) for the main effects and interactions of variables. The sperm quality parameters were then compared using a Tukey HSD test. In experiment 2, data from both storage temperature conditions (4 or 15 °C) were analyzed through the logistic regression to compare the bacterial load and pregnancy rate. In both experiments, a *p*-value of <0.05 was deemed statistically significant. Results were expressed as the model-derived mean ± standard error of the mean (SEM).

## 3. Results

### 3.1. Experiment 1

#### 3.1.1. Sperm Motility Characteristics (Motility and Kinematics)

The results given in Table 1 indicate that the progressive and total motility of rabbit sperm were significantly influenced by sericin treatment, storage temperature, and storage duration. Interactions were observed between storage duration and storage temperature for both progressive and total motility, as well as between sericin treatment and storage temperature for total motility. As shown in Table 1, storage temperature significantly affected all sperm kinematic variables (VCL, VSL, VAP, LIN, STR, WOB, ALH, and BCF). The sperm VCL, VSL, VAP, ALH, and BCF were significantly influenced by sericin treatment, while VCL, VAP, STR, WOB, and BCF were significantly affected by storage duration. The interaction between storage duration and storage temperature was observed for VCL, VAP, and BCF. The rest of the sperm kinematic variables did not show interactions between sericin treatment and storage temperature or between sericin treatment and storage duration. However, WOB showed an interaction with storage temperature, sericin treatment, and storage duration.

#### 3.1.2. Sperm Viability, Plasma Membrane Integrity, and Acrosome Integrity

**Figure 1:**
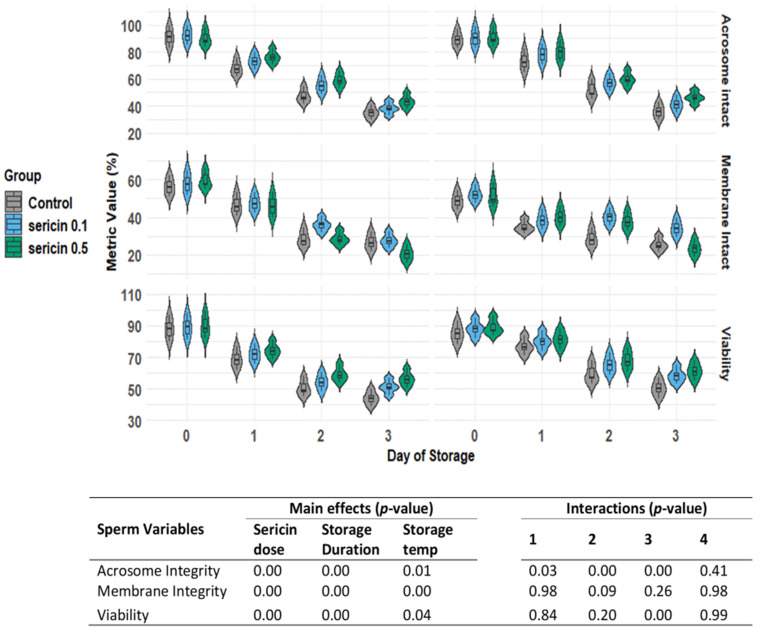
Violin plot illustrating the rabbit sperm acrosome integrity, sperm plasma membrane integrity, and sperm viability over 72 h is shown along the x-axis, with storage temperatures of 4 or 15 °C indicated above the x-axis and the percentage of each parameter represented along the y-axis. The treatment groups (control and sericin 0.1% and 0.5%) are distinguished by the colors on the left margin of the graph. The width of each violin indicates the distribution of observed values for the variable on each day. Additionally, the table represents the main effects (sericin dose, storage time, and storage temperature) and interactions [sericin dose × storage temperature (1), sericin dose × storage duration (2), storage duration × storage temperature (3), and sericin dose × storage duration × storage temperature (4)].

Based on the results for acrosome integrity, membrane integrity, and sperm viability staining, significant effects of sericin treatment, storage temperature, and storage duration were observed. Interactions were found between sericin treatment and storage temperature, sericin treatment and storage duration, and storage temperature and storage duration concerning membrane integrity. However, interactions were non-significant for acrosome integrity, although an interaction between storage temperature and storage duration was found for the sperm viability rate (Figure 1).

#### 3.1.3. Bacterial Load

**Figure 2:**
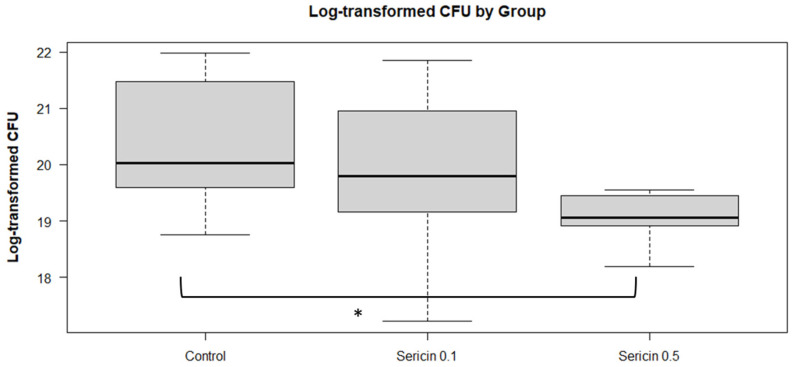
The box plot represents the log-transformed CFU counts in rabbit semen across different treatment groups stored at 15 °C. An asterisk (*) indicates a statistically significant difference between the control and the sericin 0.5 group (*p* < 0.05, Tukey-adjusted).

The estimated marginal means of the log CFU count, presented in Figure 2, indicates that the log CFU count for the control group was significantly different from that of the sericin 0.5 group (*p* = 0.0463). However, comparisons between the control group and the sericin 0.1 group (*p* = 0.6538), or between sericin treatment groups (sericin 0.1 vs. sericin 0.5, *p* = 0.2603), did not show any statistical difference.

#### 3.1.4. In Vivo Fertility

**Figure 3:**
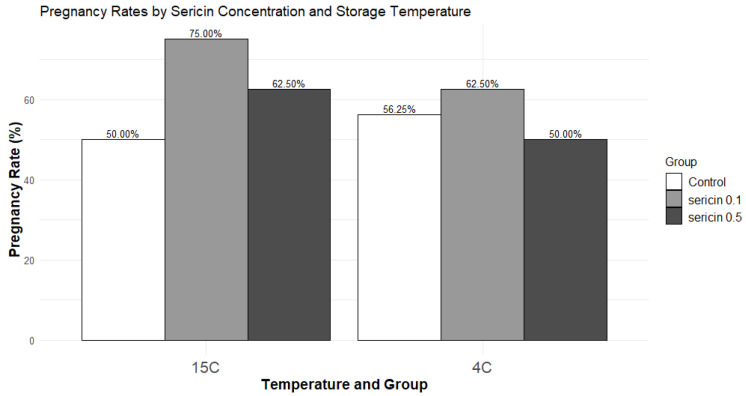
Effect of sericin concentration and storage temperature on pregnancy rates in rabbit does inseminated with semen incubated for 72 h at 4 or 15 °C.

A numerically higher percentage of rabbit does became pregnant when inseminated by semen treated with sericin 0.1% (75%) compared to the control or sericin 0.5% groups (50% and 62.5%, respectively), particularly at 15 °C. Logistic regression analysis showed no significant effect of sericin concentrations (0.1 or 0.5%) or storage temperatures (4 or 15 °C) and their interaction on the likelihood of achieving pregnancy (Figure 3).

## 4. Discussion

The present study results show that sericin supplementation appears to maintain the progressive and total motility of sperm stored at 4 °C and 15 °C over a period of three days. However, a significant decrease in sperm quality parameters was observed on days 2 and 3 of storage at these temperatures. This sharp decline is consistent with previous research attributing the vulnerability of rabbit sperm to oxidative damage, primarily due to the high content of polyunsaturated fatty acids and limited tolerance to chilling [27,28]. Several additives, including caffeine, gelatin, quercetin, methionine, and various antioxidants, have been investigated to prolong the storage time and fertility of rabbit semen at 4 or 15 °C. These substances have shown promising results in maintaining sperm motility and other quality parameters, extending sperm lifespan by up to 3–4 days [29,30,31,32].

The sustained pattern motility (both total and progressive) of rabbit sperm stored at 15 °C was observed with sericin treatment (0.1%) over an extended period (72 h). This effect is attributed to the bioactive properties of sericin, which enhance sperm resilience to cold stress [33]. Numerous reports describe the beneficial effects of sericin with the same dose level during cryopreservation, while maintaining the motility in post-thaw semen has been documented in rooster sperm using a smaller dose of sericin [22]. Additionally, the consistent pattern of sperm motility in sericin-treated samples indicates that sericin may be involved in the metabolic activity of sperm, although further investigations are needed.

The relationship between sperm kinematics and fertility outcomes has long been recognized in rabbits. This study posited that supplementing sericin could enhance sperm kinematics, thereby potentially improving fertility outcomes [30]. A similar trend in rabbit sperm kinematics variables, as observed in motility, was observed by sericin treatment in response to storage temperature and duration. These results suggest that sericin exerts a protective effect on sperm kinematics during storage at cooler temperatures by providing antioxidant and antibiotic properties, thereby helping rabbit sperm sustain their viability for a longer duration. This aligns with the results of Merati et al. [34] who reported that sericin reduced MDA production and improved velocity parameters in frozen–thawed ram semen, comparable to the effects of minocycline.

One major challenge encountered in rabbit liquid semen storage is the “dilution effect”, whereby extending semen beyond a certain threshold leads to alterations in sperm plasma membrane structure and function [35]. While some reports suggest that increased dilution rates provide substantial nutrients for extended storage, the dilution effect can reduce the availability of plasma proteins for sperm, resulting in lower fertility [36]. Sericin, a biomolecule with its unique molecular properties, could be utilized as a first aid to repair damage to the plasma membrane [37], especially when addressing dilution factors. Interestingly, sericin-treated sperm exhibited slightly higher levels of membrane and acrosomal integrity from day 1 to 3 at both storage temperatures. The exact mechanism by which sericin protects rabbit sperm during refrigeration is not yet known. Assessing the antioxidants and biochemical profile of sericin-treated rabbit sperm pre- and post-exposure at cooler temperatures for different time points could reveal the underlying mechanism. Similar improvements in sperm membrane and acrosomal integrity have been reported in dairy bull and boar semen supplemented with sericin during the cryopreservation process [21,38].

In the present study, the antimicrobial activity of sericin was evaluated by selecting a storage temperature of 15 °C, which is more conducive to bacterial growth compared to refrigeration. The mean log CFU count was significantly reduced in the sericin 0.5 supplemented rabbit semen after 72 h of storage, suggesting a potential inhibitory effect of sericin treatment on microbial growth. Due to its antimicrobial effect, sericin is used in sericin-based hydrogels in wound healing [39] and cryogels for wound dressing materials [40]. Sericin could be a promising supplement in semen extenders and culture media for the maturation and fertilization of oocytes [41,42].

Interestingly, the does inseminated with sperm treated with a 0.1% sericin concentration exhibited a numerically higher pregnancy rate after 72 h of storage at 15 °C. However, logistic regression analysis did not reveal a significant effect of sericin concentration, storage temperature, or their interaction on the likelihood of achieving pregnancy. Our findings shed light on the intricate relationship between sericin concentration, temperature, and potential increase in in vivo fertility. Including a larger sample of rabbit does in insemination trials could provide a better understanding of how rabbit sperm responds to different storage conditions and durations.

## 5. Conclusions

In conclusion, treating rabbit sperm with sericin and storing it at 4 and 15 °C for 48 to 72 h has promising effects on sperm quality. Furthermore, the antibacterial role of sericin during liquid semen storage at lower temperatures has been elucidated. Treatment with sericin could be a potential way to enhance pregnancy outcomes when rabbit sperm is refrigerated for extended durations.

## Figures and Tables

**Table 1:** Rabbit sperm motility and kinematics stored at 4 and 15 °C for a duration of 72 h in semen extender supplemented with sericin.

Parameters	Treatment	Time of Storage									Main Effects (*p*-Value)			Interactions (*p*-Value)			
		0 h		24 h		48 h		72 h		P SEM	Sericin Dose	Storage Duration	Storage Temperature	1	2	3	4
		4 °C	15 °C	4 °C	15 °C	4 °C	15 °C	4 °C	15 °C								
Progressive motility (%)	Control	53.4	52.0	35.2	36.1	16.4	30.1	5.8	10.4	2.10	0.00	0.00	0.00	0.20	0.48	0.00	0.73
	Sericin 0.1	55.2	53.2	38.8	45.8	17.8	34.6	8.7	19.2								
	Sericin 0.5	54.4	52.2	38.9	43.6	18.6	33.2	12.9	15.1								
Total motility (%)	Control	85.4	84.4	56.2	62.4	48.0	56.0	17.6	32.0	2.33	0.00	0.00	0.00	0.00	0.16	0.00	0.73
	Sericin 0.1	86.2	86.0	62.8	72.0	53.6	63.6	28.0	41.8								
	Sericin 0.5	87.1	85.0	65.8	64.8	57.8	58.0	31.8	35.6								
VCL (μm/s)	Control	102.8	94.4	85.6	89.0	77.6	82.6	74.6	59.8	3.20	0.00	0.00	0.02	0.45	0.06	0.00	0.68
	Sericin 0.1	101.8	95.4	97.4	102	87.4	85.8	79.6	65.1								
	Sericin 0.5	103.4	106.6	100.8	105.1	96 2	97.8	81.6	69.6								
VSL (μm/s)	Control	50.1	49.5	36.1	34.4	26.2	31.5	21.7	19.2	1.99	0.00	0.00	0.25	0.17	0.91	0.06	0.58
	Sericin 0.1	52.4	51.9	40.5	38.9	29.2	33	23.8	22.7								
	Sericin 0.5	52.1	50.5	43.8	35	33.4	31.5	23.2	23								
VAP (μm/s)	Control	63.0	61.8	52.0	41.0	35.4	38.3	27.0	21.8	2.07	0.05	0.00	0.00	0.24	0.36	0.00	0.75
	Sericin 0.1	63.2	59.1	54.8	47.0	39.0	39.1	29.4	25.2								
	Sericin 0.5	63.2	60.8	56.8	40.7	41.6	40.6	30.4	22.4								
LIN (%)	Control	41.7	41.2	31.2	32.7	25.6	26.9	24.3	21.8	1.78	0.23	0.00	0.09	0.40	0.79	0.71	0.99
	Sericin 0.1	42.6	41.1	32.2	32.5	29	27.7	25.3	23.1								
	Sericin 0.5	42.7	40.5	33.4	31.1	30.9	28.3	25.6	22.8								
STR (%)	Control	72.3	65.2	72.2	70.9	66.7	66.0	64.2	56.2	2.19	0.98	0.00	0.00	0.87	0.98	0.24	0.73
	Sericin 0.1	71.2	68.5	71.5	70.3	65.9	62.8	63.4	56.4								
	Sericin 0.5	70.3	70.2	71.5	68.1	67.6	63.7	62.6	57.4								
WOB (%)	Control	46.4	42.7	43.7	45.9	39.1	44.9	33.0	38.8	1.80	0.37	0.00	0.03	0.57	0.65	0.93	0.00
	Sericin 0.1	48.9	44.8	43.3	45.8	39.5	43.5	35.2	39.7								
	Sericin 0.5	49.9	46.7	41.9	44.1	42.1	42.5	36.2	39.2								
ALH (μm)	Control	4.1	4.3	3.5	3.5	2.9	2.8	2.2	2.7	0.39	0.03	0.00	0.47	0.21	0.36	0.92	0.99
	Sericin 0.1	4.3	4.1	3.8	3.7	3.3	3.4	2.7	2.9								
	Sericin 0.5	4.4	4.1	4.1	3.4	3.6	3.2	3.1	2.9								
BCF (Hz)	Control	11.5	11.6	9.9	10.6	8.4	5.8	4.7	4.5	0.54	0.00	0.00	0.00	0.35	0.28	0.00	0.08
	Sericin 0.1	12.7	12.1	11.3	12.8	9.8	8.6	8.5	5.2								
	Sericin 0.5	12.8	12.5	11.1	13.2	10.1	8.9	9.2	6.5								

*p* = 0.05 showing significance; VCL = curvilinear velocity; VSL = straight linear velocity; VAP = average path velocity; LIN = linearity; STR = sperm track straightness; WOB = wobble; ALH = amplitude of lateral head displacement; BCF = beat cross-frequency; 1 = sericin dose and storage temperature interaction; 2 = sericin dose and storage duration interaction; 3 = storage duration and storage temperature interaction; and 4 = sericin dose, storage duration, and storage temperature interaction.

## Data Availability

All the data and materials will be available upon reasonable request from the corresponding author.
